# Physical Activity Across Adulthood and Bone Health in Later Life: The 1946 British Birth Cohort

**DOI:** 10.1002/jbmr.3607

**Published:** 2018-12-10

**Authors:** Stella G Muthuri, Kate A Ward, Diana Kuh, Ahmed Elhakeem, Judith E Adams, Rachel Cooper

**Affiliations:** ^1^ MRC Unit for Lifelong Health and Ageing at UCL London UK; ^2^ MRC Lifecourse Epidemiology Unit University of Southampton Southampton UK; ^3^ MRC Nutrition and Bone Health Research Group Cambridge UK; ^4^ MRC Integrative Epidemiology Unit at University of Bristol Bristol UK; ^5^ Manchester Academic Health Science Centre Central Manchester University Hospitals NHS Foundation Trust, Manchester Royal Infirmary Manchester UK

**Keywords:** EXERCISE, EPIDEMIOLOGY, AGING, DXA, BONE QCT

## Abstract

Leisure‐time physical activity (LTPA) is widely recommended for the prevention of osteoporosis and fractures in older populations. However, whether the beneficial effects of LTPA on bone accumulate across life and are maintained even after reduction or cessation of regular PA in later life is unknown. We examined whether LTPA across adulthood was cumulatively associated with volumetric and areal bone mineral density (vBMD, aBMD) at ages 60 to 64 and whether associations were mediated by lean mass. Up to 1498 participants from the Medical Research Council National Survey of Health and Development were included in analyses. LTPA was self‐reported at ages 36, 43, 53, and 60 to 64, and responses summed to generate a cumulative score (range 0 = inactive at all four ages to 8 = most active at all four ages). Total and trabecular vBMD were measured at the distal radius using pQCT and aBMD at the total hip and lumbar spine (L1 to L4) using DXA. Linear regression was used to test associations of the cumulative LTPA score with each bone outcome. After adjustment for height and weight, a 1‐unit increase in LTPA score (95% CI) in men was associated with differences of 1.55% (0.78% to 2.31%) in radial trabecular vBMD, 0.83% (0.41% to 1.25%) in total hip aBMD, and 0.97% (0.44% to 1.49%) in spine aBMD. Among women, positive associations were seen for radial trabecular vBMD and total hip aBMD, but only among those of greater weight (LTPA × weight interaction *p* ≤ 0.01). In men, there was evidence to suggest that lean mass index may partly mediate these associations. These findings suggest that there are cumulative benefits of LTPA across adulthood on BMD in early old age, especially among men. The finding of weaker associations among women suggests that promotion of specifıc types of LTPA may be needed to benefit bone health in women. © 2018 The Authors. *Journal of Bone and Mineral Research* Published by Wiley Periodicals, Inc.

## Introduction

Regular leisure‐time physical activity (LTPA) is widely recommended for the prevention of osteoporosis, falls, and fractures in older populations.^1^ However, the intensity and quantity of LTPA that people achieve declines with increasing age,^2^, ^3^, ^4^, ^5^ and a high proportion of older adults do not meet recommended levels.^6^, ^7^, ^8^, ^9^

Prospective studies of children, adolescents, and younger adults suggest that participation in regular physical activity in earlier life may be beneficial for bone structure and strength in early to mid‐adulthood.^10^, ^11^, ^12^ However, most studies examining whether the effects of regular PA on bone outcomes extend into older age are cross‐sectional and/or have relied on long‐term recall of prior PA levels.^13^, ^14^, ^15^ Because of a lack of prospective studies across adulthood, it remains unclear whether the beneficial effects of PA on bone accumulate across life and are maintained even after reduction or cessation of regular LTPA in old age.

In addition to general recommendations on PA issued for the benefit of overall health, there is evidence to suggest that higher impact activities are particularly beneficial for bone health.^16^, ^17^ This is because higher levels of loading lead to adaptations in bone that maintain strength to withstand the loads; this is the premise of the mechanostat theory.^18^ For example, accelerometer‐measured PA producing higher vertical impacts ≥3.9*g* (elicited by activities such as jumping and running) has been found to be positively related to BMD in adolescents^16^ and premenopausal women.^17^ Although recent work has demonstrated that few older adults are regularly achieving PA of this level of vertical impact,^19^ PA with impact <3.9*g* may also indirectly benefit bone health^20^ via a positive effect on the maintenance of muscle mass.^18^ Though most studies examining the association between PA earlier in life and bone outcomes in older age have accounted for body weight as a potential confounder,^12^, ^13^, ^14^, ^15^, ^21^, ^22^ only one has evaluated the mediating role of lean mass; this study was small (*n* = 282) and included women only.^23^

We used data from the Medical Research Council (MRC) National Survey of Health and Development (NSHD), a British birth cohort study with longitudinal observations of physical activity across adulthood, to address these research gaps. We examined whether LTPA across adulthood is cumulatively associated with bone health in early old age and assessed the extent to which any associations were mediated by lean mass.

## Participants and Methods

### Participants

The MRC NSHD is comprised of 5362 singleton births that occurred in England, Scotland, and Wales in one week in March 1946. Participants have been prospectively followed up to 24 times since birth.^24^, ^25^ At age 60 to 64 years, study members known to be alive and living in England, Scotland, or Wales were invited for assessment at one of six clinical research facilities (CRFs) or to be visited at home by a research nurse. Of the 2856 invited, 2229 were assessed of whom 1690 attended a CRF. Ethical approval for this data collection was obtained from the Central Manchester Research Ethics Committee (07/H1008/245) and the Scottish A Research Ethics Committee (08/MRE00/12). Written informed consent was provided by each participant.

### Bone densitometry and body composition assessments at age 60 to 64

pQCT scans of the nondominant distal (4%) and midshaft (50%) radius were undertaken in five CRFs using XCT 2000 (Stratec Medizintechnik, Pforzheim, Germany) scanners. DXA measurements at all CRFs were performed using QDR 4500 Discovery (Hologic Inc, Bedford, MA, USA) scanners. Lean and fat mass were assessed from whole‐body scans, and for analysis the indices derived by dividing each measure by height; lean mass index (LMI; kg/m^2^) and fat mass index (FMI; kg/m^1.2^). Scans of the proximal femur and spine were obtained for measurement of areal bone mineral density (aBMD). Detailed descriptions of scan acquisition including quality assurance have previously been published.^26^, ^27^

Of those attending a CRF, 1685 (792 men) underwent a DXA scan and 1355 (658 men) had a pQCT scan. Of the range of measures ascertained, we decided a priori to focus on BMD measurements in those sites prone to fracture: total hip and lumbar spine (L1 to L4) aBMD and trabecular and total radius volumetric BMD (vBMD).

### Assessment of physical activity

Participation in LTPA was ascertained during nurse interviews at ages 36, 43, 53, and 60 to 64. At age 36, a modified Minnesota LTPA questionnaire was used to assess how often in the previous month study members participated in 27 different activities. At age 43, participants were asked whether they had taken part in any sports, vigorous leisure activities, or exercise in the previous year and the duration (in months) and frequency of participation. At ages 53 and 60 to 64, study members reported whether they participated in any sports, vigorous leisure activities, or exercise in the previous 4 weeks.^28^ At each age, participants were grouped as inactive (no participation in LTPA), moderately active (1 to 4 times/month), or most active (≥5 times/month), coded 0, 1, and 2, respectively. As our primary focus was cumulative exposure to LTPA, we then summed LTPA responses at each age to generate a total score ranging from 0 = inactive at all four ages to 8 = most active at all four ages.

### Covariates

Potential confounders and mediators were identified a priori based on existing literature.^29^, ^30^, ^31^

Weight (kg) and height (cm) were measured at age 60 to 64 by a trained nurse. Body composition at this age was measured from supine whole‐body DXA scans, and whole‐body (excluding head) lean mass index (LMI; kg/height [m^2^] and fat mass index (FMI; kg/height [m^1.2^] were calculated.^26^ Smoking status was reported up to age 60 to 64 and categorized as never, ex‐, and current smoker. Occupational class at age 53 years was categorized according to the Registrar General's social classification and collapsed into two groups: nonmanual (I, II, IIINM) or manual (IIIM, IV, V). At age 60 to 64, participants reported any longstanding illness or health problems that had lasted, or were expected to last for 6 months or more. In women, information on menstrual irregularity, month and year of last menstrual cycle or any operation to remove the uterus or ovaries, and monthly hormone‐replacement therapy use was ascertained during nurse interviews at ages 43, 53, and 60 to 64 and in annual postal questionnaires between ages 47 and 54 years (inclusive) and at age 57. This was used to define type of menopause (ie, natural versus surgical). Timing of menopause was calculated as age since birth until periods ceased.^31^

### Statistical analysis

Linear regression models were used to examine the associations between the cumulative LTPA score and each bone outcome in sex‐stratified models. All outcomes were transformed using natural logarithms and results presented as percentage differences.^32^ Formal tests of sex interaction were performed by including sex by LTPA interaction terms in models including both men and women. Tests of deviation from linearity in the cumulative LTPA score were performed by including quadratic terms, but no evidence of this was found. Models were first adjusted for current weight and height. We tested for interaction between the cumulative LTPA score and current weight as associations have previously been found to differ by body composition;^26^, ^30^ where this was evident, subsequent analyses for these outcomes were stratified by weight. Models were then adjusted for smoking status, occupational class, and long‐term illness, plus type and age at menopause in women.

As the cumulative adulthood LTPA score used in these analyses assigns equal weight to LTPA at each of the four ages, we also applied a structured modeling approach^28^, ^33^ to test whether accumulation models that allowed for effect sizes to vary by age fitted the data as well as those that assumed similar effect sizes at each age or sensitive period models. Partial *F* tests were used to compare each of these models with a fully saturated model, which assumed that all possible trajectories of LTPA across adulthood were associated with bone outcomes with adjustments made for current weight and height. Large *p* values indicated that the nested model fit the data as well as the saturated model.

Where overall associations between the cumulative LTPA score and bone outcomes were observed, to explore whether these were mediated by lean mass, fully adjusted models were rerun with the inclusion of LMI instead of current weight. As fat and lean mass are highly correlated and lean mass accrual often occurs in response to fat mass accrual,^34^ these models were also adjusted for FMI, and the degree of attenuation in estimates was compared before and after adjustment for LMI. Where the cumulative adulthood LTPA score remained associated with an outcome after adjustment for height, LMI, FMI, and covariates at the 5% level, structural equation modeling was then used to estimate direct and indirect (through lean mass) paths between the cumulative LTPA score and each bone outcome and the percentage of the association mediated by LMI was estimated.

As the cumulative LTPA score assigns people with different patterns of participation in LTPA across adulthood the same score, we also investigated whether change in LTPA was associated with each bone outcome. For this analysis, change in LTPA was determined based on reports of LTPA at ages 36 and 60 to 64, with participants classified as inactive at both ages; active at age 60 to 64, but inactive at age 36; inactive at age 60 to 64, but active at age 36; or active at both ages. Regression models were run with adjustments made for the same covariates as described above for the cumulative LTPA score.

To reduce potential bias because of missing data, missing values for covariates (ie, smoking status, occupational class, long‐term illness, LMI, FMI, plus type and timing of menopause in women) were imputed using multiple imputation by chained equations. Analyses were performed across 20 imputed datasets and combined using Rubin's rules.^35^

Sensitivity analyses were run in which the main analyses were rerun restricted to the sample of participants who had complete data on exposure variables, bone outcomes, and covariates for comparison with those run on imputed data. Findings were similar, so analyses using imputed data are presented.

All analyses were performed using STATA version 14.2 (Stata Corp., Inc., College Station, TX, USA).

## Results

Of the 1685 participants who underwent a DXA and/or pQCT scan at ages 60 to 64, 1656 had at least one bone outcome measure and LTPA at any age in adulthood. Characteristics of this sample stratified by sex are presented in Table 1.

**Table 1:** Characteristics of the NSHD Sample With at Least One Bone Measure and at Least One Leisure‐Time Physical Activity (LTPA) Measure

	Men		Women		
	*N*	Mean (SD) or %	*N*	Mean (SD) or %	*p* Value
Bone outcomes at age 60 to 64					
Trabecular radius vBMD (mg/cm^3^), 4% distal radius	657	205.9 (42.8)	687	172.0 (42.7)	<0.001
Total density vBMD (mg/cm^3^), 4% distal radius	657	390.3 (66.6)	687	329.4 (70.5)	<0.001
Total hip aBMD (g/cm^2^)	780	1.00 (0.15)	855	0.86 (0.13)	<0.001
Spine L1 to L4 aBMD (g/cm^2^)	790	1.05 (0.19)	860	0.94 (0.16	<0.001
LTPA at each age in adulthood:					
36 years					0.001
Inactive	197	27.6	284	35.8	
Moderately active (1 to 4 times/ month)	203	28.4	219	27.6	
Most active (≥5 times/ month)	314	44.0	290	36.6	
43 years					0.007
Inactive	310	41.6	397	48.1	
Moderately active (1 to 4 times/ month)	195	26.1	218	26.4	
Most active (≥5 times/ month)	241	32.3	211	25.5	
53 years					0.110
Inactive	287	38.8	357	42.3	
Moderately active (1 to 4 times/ month)	172	23.2	161	19.2	
Most active (≥5 times/ month)	281	38.0	321	38.3	
60 to 64 years					0.580
Inactive	462	59.8	488	57.5	
Moderately active (1 to 4 times/ month)	115	14.9	140	16.5	
Most active (≥5 times/ month)	196	25.4	221	26.0	
Cumulative LTPA score	647	3.69 (2.39)	745	3.41 (2.36)	0.033
LTPA at ages 36 and 60 to 64					<0.001
Inactive at both ages	159	22.7	191	24.6	
Inactive at 36, active at 60 to 64	35	5.0	88	11.3	
Active at 36, inactive at 60 to 64	264	37.7	253	32.5	
Active at both ages	242	34.6	246	31.6	
Covariates (at age 60 to 64 unless otherwise specified)					
Height (cm)	791	175.3 (6.4)	865	162.1 (5.8)	<0.001
Weight (kg)	791	85.3 (13.1)	865	72.3 (14.1)	<0.001
Fat mass index (kg/m^1.2^)	746	12.1 (3.6)	811	16.2 (5.1)	<0.001
Lean mass index (kg/m^2^)	746	17.5 (2.0)	811	14.2 (1.8)	<0.001
Occupational class at age 53					<0.001
Nonmanual	569	72.5	692	80.3	
Manual	216	27.5	170	19.7	
Smoking status					<0.001
Never	209	28.9	305	38.3	
Ex‐smoker	442	61.1	420	52.8	
Current smoker	73	10.1	71	8.9	
Longstanding illness or health problem					0.840
No	618	78.1	670	77.7	
Yes	173	21.9	192	22.3	
Age (years) at natural menopause	n/a		520	51.9 (3.8)	
Age (years) at hysterectomy	n/a		190	44.5 (6.6)	
Type of menopause; *N* (%)	n/a		847		
Natural menopause				652 (77.0)	
Hysterectomy				195 (23.0)	

vBMD = volumetric bone mineral density; areal bone mineral density = areal bone mineral density.

In men, a higher cumulative LTPA score was linearly associated with higher radius trabecular vBMD and higher aBMD at the hip and lumbar spine in unadjusted models (Table 2: model 1). Adjustment for current height and weight slightly attenuated differences in aBMD at the hip and spine (Table 2: model 2), whereas estimates for radius trabecular vBMD were partially attenuated after additional adjustment for other covariates (Table 2: model 3). Results comparing different life course models supported these findings, indicating that the accumulation model allowing for differences in effect size at each age fit the data as well as the fully saturated model for trabecular vBMD, hip aBMD, and spine aBMD (*p* values from partial *F* tests = 0.13, 0.34, and 0.28, respectively).

**Table 2:** Percentage Differences in DXA‐ and pQCT‐Derived Outcomes per 1 Unit Increase in a Cumulative LTPA Score^a^

	Radius trabecular vBMD^b^		Radius total density vBMD		Total hip aBMD^b^		Spine L1 to L4 aBMD	
	% diff (95% CI)	*p* Value for trend	% diff (95% CI)	*p* Value for trend	% diff (95% CI)	*p* Value for trend	% diff (95% CI)	*p* Value for trend
Men	*N* = 533		*N* = 535		*N* = 638		*N* = 645	
Model 1	1.51 (0.74 to 2.28)	<0.001	0.03 (−0.59 to 0.65)	0.93	0.89 (0.42 to 1.37)	<0.001	1.02 (0.47 to 1.57)	<0.001
Model 2	1.55 (0.78 to 2.31)	<0.001	0.04 (−0.58 to 0.66)	0.9	0.83 (0.41 to 1.25)	<0.001	0.97 (0.44 to 1.49)	<0.001
Model 3	1.43 (0.64 to 2.22)	<0.001	−0.04 (−0.69 to 0.6)	0.89	0.88 (0.45 to 1.32)	<0.001	1.08 (0.54 to 1.61)	<0.001
Women	*N* = 589		*N* = 590		*N* = 737		*N* = 742	
Model 1	−0.81 (−1.71 to 0.09)	0.078	−0.66 (−1.4 to 0.07)	0.08	−0.19 (−0.65 to 0.26)	0.410	0.06 (−0.47 to 0.59)	0.83
Model 2	−0.16 (−1.09 to 0.76)^d^	0.73	−0.20 (−0.95 to 0.55)	0.60	0.50 (0.09 to 0.91)^d^	0.017	0.48 (−0.04 to 1.00)	0.07
	0.09 (0.03 to 0.16)^e^	0.003^*^			0.03 (0.01 to 0.06)^e^	0.013^*^		
Model 3^c^	−0.30 (−1.26 to 0.65)^d^, ^f^	0.53	−0.27 (−1.05 to 0.51)	0.50	0.37 (−0.05 to 0.79)^d^, ^f^	0.08	0.40 (−0.13 to 0.93)	0.14
	0.10 (0.03 to 0.16)^e^	0.003^*^			0.04 (0.01 to 0.06)^e^	0.011^*^		
*p* for sex interaction								
Model 1		0.0001		0.16		0.0013		0.013
Model 2		0.009^**^		0.43		0.070^**^		0.2
Model 3		0.011^**^		0.43		0.090^**^		0.2

vBMD = volumetric bone mineral density; areal bone mineral density = areal bone mineral density.

^a^ Cumulative LTPA score range: 0 = Inactive at all 4 ages to 8 = Most active at all 4 ages (36, 43, 53, and 60 to 64). Model 1: unadjusted; Model 2: current height and weight; Model 3: model 2 + current smoking status, occupational class and long‐term illness.

^b^ In women, interaction between LTPA x weight *p* ≤ .01.

^c^ Same model adjustments as for men plus type and timing of menopause.

^d^ Effect estimate for a 1‐unit increase in the LTPA score for women of mean weight (72.3 kg).

^e^ LTPA score x weight interaction term.

**Figure 1:**
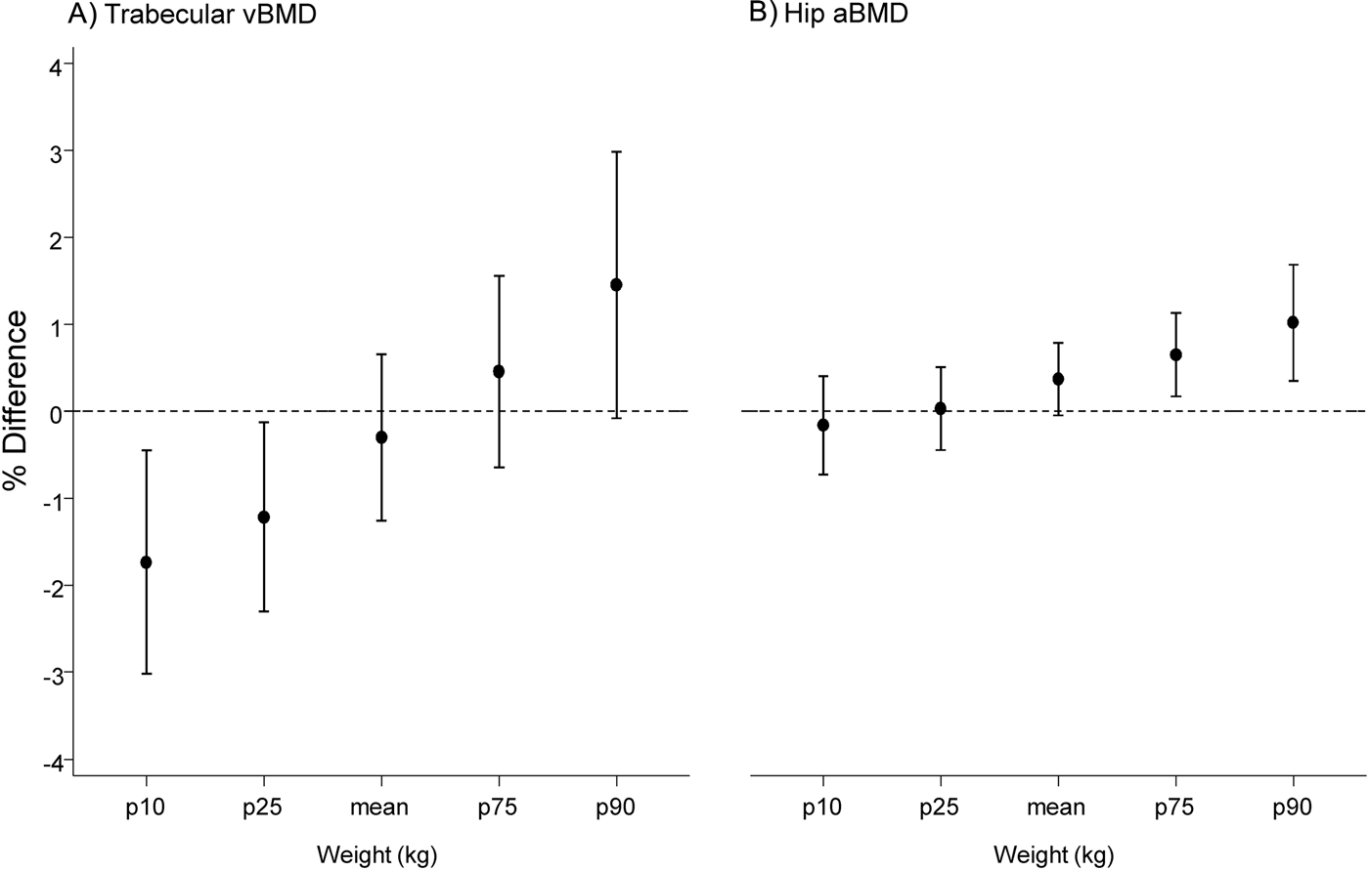
Percentage differences in (*A*) radius trabecular volumetric bone mineral density (vBMD) and (*B*) hip areal bone mineral density (aBMD) per 1 unit increase in a cumulative leisure‐time physical activity (LTPA) score by weight among women. Note: Cumulative score range: 0 = inactive at all four ages to 8 = most active at all four ages (36, 43, 53, and 60 to 64). *P* = percentile. p10 (57.4 kg); p25 (62.8 kg); mean (72.3 kg); p75 (80.2 kg); p90 (90.6 kg). Effect estimates presented are adjusted for covariates.

^f^ Estimates at the 10th, 25th, 75th, and 90th percentiles are shown in Fig.  1.

^*^ *p* value for LTPA score x weight interaction.

^**^ *p* value for LTPA score x sex x weight interaction.

In women, there were no associations between the cumulative LTPA score and any of the bone outcomes in unadjusted models (*p* for sex interactions ≤.01; Table 2: model 1). However, there were interactions between the LTPA score and weight for trabecular radius vBMD and hip aBMD (*p* = 0.003 and *p* = 0.013, respectively); associations between higher cumulative LTPA scores and higher levels of these two outcomes were only evident in women with higher body weight, whereas inverse associations were observed among those with lower body weight for trabecular radius vBMD (Table 2: model 2). Adjustment for covariates attenuated these estimates (Table 2: model 3) and findings from these models are presented in Fig. 1A and 1B. These findings were confirmed using the structured approach, which showed that the accumulation model assuming similar effect sizes at each age was the best‐fitting model for trabecular radius vBMD and hip aBMD (*p* values from *F* tests comparing the accumulation model to a fully saturated model =.27 and =.62, respectively) for those in the highest fifth of weight.

When we examined whether the associations found in men were mediated by lean mass, we observed that adjustment for LMI caused some reduction in mean percentage differences (Supplemental Table  1). Results from path analysis showed that the percentage of the association mediated by LMI was 12.6%, 27.7%, and 23.3% for trabecular radius vBMD, hip aBMD, and lumbar spine aBMD, respectively. In women, associations with hip aBMD were also largely attenuated by LMI. By contrast, LMI had little impact on the association between LTPA score and trabecular radius vBMD.

Consistent with findings from models of the cumulative LTPA score, when different patterns of LTPA between ages 36 and 60 to 64 were examined, we found that men who were active at both ages had the highest levels of radius trabecular vBMD, hip aBMD, and lumbar spine aBMD even after adjustment for covariates (Table 3: model 3). Those who were active at age 36, but inactive at age 60 to 64 also had higher mean levels of hip aBMD than those who were inactive at both ages; however, there was no evidence that the group who was inactive at age 36, but active at age 60 to 64 was different from the group who were inactive at both ages. In women, associations with radius trabecular vBMD and hip aBMD differed by weight at age 60 to 64 (*p* value for interaction = 0.006 and 0.001, respectively); associations were stronger for those who were heavier and participated in LTPA at ages 36 and 60 to 64 compared to those who were inactive at both ages (Table 3: model 2). Adjustment for covariates attenuated most of these estimates, but associations remained for trabecular radius vBMD and hip aBMD where positive associations were more pronounced among heavier women in the group who were active at both ages (Table 3: model 3; Supplemental Fig.  1).

**Table 3:** Percentage Differences in DXA‐ and pQCT‐Derived Outcomes by Changes in Leisure‐Time Physical Activity (LTPA) Between Ages 36 and 60 to 64 (Inactive at Both Ages as the Reference Category)^a^

	Men				Women				
	Inactive at 36, active at 60 to 64	Active at 36, inactive at 60 to 64	Active at both ages	*p* value*	Inactive at 36, active at 60 to 64	Active at 36, inactive at 60 to 64	Active at both ages	*p* Value*	*p* Sex int
	% diff (95% CI)	% diff (95% CI)	% diff (95% CI)		% diff (95% CI)	% diff (95% CI)	% diff (95% CI)		
Trabecular radius vBMD									
*N*, men = 584; women = 616									
Model 1	−0.83 (−9.30 to 7.63)	3.87 (−0.71 to 8.45)	8.74 (4.07 to 13.42)	0.0001	−3.68 (−11.02 to 3.67)	−2.11 (−7.78 to 3.55)	−3.99 (−9.66 to 1.68)	0.550	0.007
Model 2^b^	−0.61 (−9.03 to 7.82)	3.37 (−1.19 to 7.93)	8.64 (4.00 to 13.29)	0.0001	−1.43 (−8.84 to 5.98)^d^ 0.52 (0.02 to 1.01)^e^	−0.52 (−6.31 to 5.28)^d^ 0.30 (‐0.06 to 0.66)^e^	−0.86 (−6.72 to 4.99)^d^ 0.70 (0.29 to 1.11)^e^	0.980 0.006**	0.028***
Model 3^b^,^c^	−1.49 (−10.01 to 7.03)	3.08 (−1.50 to 7.67)	8.06 (3.30 to 12.82)	0.003	−2.31 (−9.77 to 5.16)^d^,^f^	−0.89 (−6.70 to 4.93)^d^,^f^	−1.70 (−7.67 to 4.27)^d^,^f^	0.92	0.007***
					0.48 (−0.02 to 0.97)^e^	0.29 (−0.08 to 0.65)^e^	0.72 (0.31 to 1.14)^e^	0.005**	
Total density vBMD									
*N*, men = 586; women = 617									
Model 1	−5.90 (−12.79 to 0.99)	−1.26 (−4.98 to 2.46)	−1.46 (−5.26 to 2.34)	0.41	1.55 (−4.43 to 7.53)	−2.42 (−7.03 to 2.19)	−2.62 (−7.24 to 1.99)	0.380	0.29
Model 2	−5.77 (−12.67 to 1.13)	−1.43 (−5.17 to 2.30)	−1.49 (−5.29 to 2.31)	0.43	4.54 (−1.49 to 10.57)	0.17 (−4.50 to 4.84)	0.47 (−4.28 to 5.21)	0.43	0.28
Model 3^c^	−6.66 (−13.64 to 0.32)	−1.71 (−5.45 to 2.04)	−2.15 (−6.04 to 1.75)	0.29	4.22 (−1.88 to 10.32)	0.24 (−4.45 to 4.93)	0.34 (−4.53 to 5.21)	0.51	0.28
Hip aBMD									
*N*, men = 691; women = 769									
Model 1	0.81 (−4.53 to 6.15)	4.51 (1.63 to 7.39)	3.41 (0.48 to 6.35)	0.016	−1.18 (−5.01 to 2.64)	0.27 (−2.57 to 3.11)	−1.08 (−3.93 to 1.77)	0.710	0.13
Model 2^b^	−0.49 (−5.22 to 4.25)	3.20 (0.64 to 5.75)	2.87 (0.27 to 5.47)	0.04	−1.18 (−2.18 to 4.54)^d^	1.76 (−0.76 to 4.28)^d^	2.79 (0.24 to 5.34)^d^	0.19	0.008***
					0.21 (−0.02 to 0.43)^e^	0.16 (0.003 to 0.32)^e^	0.37 (0.20 to 0.55)^e^	0.001**	
Model 3^b^,^c^	−0.45 (−5.23 to 4.33)	3.33 (0.77 to 5.90)	3.04 (0.39 to 5.69)	0.03	0.42 (−2.94 to 3.77)^d^,^f^	1.46 (−1.05 to 3.96)^d^,^f^	2.00 (−0.59 to 4.59)^d^,^f^	0.43	0.009***
					0.19 (−0.03 to 0.42)^e^	0.14 (−0.02 to 0.29)^e^	0.37 (0.19 to 0.54)^e^	0.001**	
Spine L1 to L4 aBMD									
*N*, men = 698; women = 774									
Model 1	−3.35 (−9.59 to 2.88)	2.33 (−1.03 to 5.68)	3.41 (−0.004 to 6.83)	0.06	−2.25 (−6.66 to 2.16)	−1.10 (−4.39 to 2.18)	−0.94 (−4.24 to 2.37)	0.780	0.2
Model 2	−4.48 (−10.42 to 1.46)	1.37 (−1.83 to 4.57)	3.00 (−0.25 to 6.25)	0.04	−0.54 (−4.78 to 3.69)	0.07 (−3.11 to 3.25)	1.57 (−1.65 to 4.79)	0.64	0.46
Model 3^c^	−3.96 (−9.97 to 2.05)	1.67 (−1.55 to 4.89)	3.33 (0.02 to 6.65)	0.04	−1.37 (−5.60 to 2.87)	−0.29 (−3.46 to 2.88)	1.14 (−2.15 to 4.42)	0.61	0.5

vBMD = volumetric bone mineral density; areal bone mineral density = areal bone mineral density.

^a^ Model 1: unadjusted; Model 2: current height and weight; Model 3: model 2 + current smoking status, occupational class and long‐term illness.

^b^ In women, interaction between change in LTPA x weight *p* ≤ 0.01.

^c^ In women, same adjustments as for men plus type and timing of menopause.

^d^ Effect estimate for women of mean weight (72.3 kg).

^e^ Change in LTPA x weight interaction term.

^f^ Estimates at the 25th (62.8 kg) and 75th (80.2 kg) percentiles are illustrated in Supplemental Fig.  1.

*p value for overall test of association.

**p value for interaction between change in LTPA and weight.

***p value for change in LTPA x weight x sex.

## Discussion

In this relatively large British birth cohort of adults aged 60 to 64 years, greater participation in LTPA across adulthood was associated with higher aBMD at the total hip and spine, as well as radius trabecular vBMD in men. Among women, there were positive associations with radius trabecular vBMD and total hip aBMD, but these were only evident among those who were heavier. Among men, adjustment for LMI resulted in some attenuation in estimates, suggesting associations observed with radius trabecular vBMD, total hip aBMD, and spine aBMD may be partly mediated by lean mass.

Our findings extend previous studies that have focused on LTPA during adulthood in relation to bone outcomes in mid‐ to later adulthood. We have shown that greater participation in LTPA over 28 years was positively associated with bone density at all sites in men, and with radius and hip bone density in heavier women in early old age. Our findings are similar to a prospective study of Norwegian men and women aged 20 to 56 years (mean age 41.5 years) at baseline, which reported that LTPA was positively associated with higher aBMD over a 22‐year follow‐up period.^12^ This study also showed that those who were moderately active or active at both baseline and follow‐up had higher aBMD than those who were least active at both assessments.^12^ Likewise, a Swedish study of adults aged 50 to 80 years at baseline, which retrospectively assessed LTPA, also showed that individuals who were classified as active at the beginning and end of the study period had lower age‐related decline in forearm aBMD than those who remained inactive over the 10‐year follow‐up period.^36^ However, evidence from retrospective studies of older adults is inconsistent with few reporting associations between physical activity across adulthood and bone outcomes assessed using DXA. Cross‐sectional studies using pQCT have also found no associations between contemporaneous physical activity and distal radius bone parameters in older men and women.^14^, ^37^, ^38^ However, consistent with our findings, prospective studies that have examined associations between LTPA during adolescence and early adulthood and bone health in early to mid‐adulthood, have reported beneficial effects of LTPA on BMD at different skeletal sites, with stronger evidence of associations in men.^10^, ^11^

We found sex differences in associations of the cumulative LTPA score with bone density at sites at risk of osteoporotic fracture, with much stronger, consistent evidence of associations in men. That the adjustment for current body size resulted in the greatest attenuation of estimates, particularly in women, suggests that our findings are partly explained by sex differences in body composition. In men, the observed associations between LTPA and bone outcomes were mainly driven by relationships between LTPA and lean mass. In women, associations of LTPA with radius trabecular vBMD and hip aBMD were stronger in those with higher body weight. Adjustment for LMI resulted in the greatest attenuation of estimates for hip aBMD, a weight‐bearing site, whereas FMI had a greater effect on radial trabecular vBMD (Supplemental Table  2). This finding is in keeping with previous cross‐sectional studies using high‐resolution pQCT, which have shown positive associations between fat mass and radius and tibia vBMD among older women, but not men.^30^, ^39^ Previous findings from NSHD have also shown that higher LTPA across adulthood was associated with lower fat mass in women and higher appendicular lean mass (in both sexes, after adjustment for fat mass).^26^ In the current study, we observed the beneficial effect of LTPA on weight‐bearing and non‐weight‐bearing bones; adjustment for LMI did not fully attenuate associations in both sexes, indicating that other pathways including those relating to sex hormones and other endocrine factors may play a role.

Another possible explanation for finding stronger associations in men than women is that the characteristics of PA (ie, type, intensity, frequency, duration, and mode) that women typically engage in across the life course may be less optimal for bone and muscle health in later life than those men engage in. Indeed, previous findings from NSHD suggest that men were more likely to participate in higher impact activities such as football and racquet sports in earlier adulthood^40^ before switching to engagement in activities of lower impact in old age,^41^ whereas women were more likely to engage in lower impact activities (eg, swimming, yoga) across adulthood.^40^, ^41^ Finally, there is the possibility that menopausal bone loss may have negated the effects of LTPA in women. Although the addition of timing of menopause did not fully attenuate the weaker associations found in women, there was some reduction in effect size (Table 2: model 3). This suggests strategies to increase LTPA in women should begin in the premenopausal period, though further work is needed to confirm this.

Our findings also suggest that associations with PA differed by weight in women; higher cumulative LTPA scores were associated with greater radial trabecular vBMD and hip aBMD among heavier women only. One possible explanation is that greater loading to the bones of heavier women during LTPA has helped to maintain or increase their bone density through a positive response to the extra loading. In contrast, Nguyen and colleagues^42^ found that the association between physical activity and femoral bone loss in postmenopausal women was more pronounced in thinner women and among those who experienced significant weight loss, but no such effects were observed among those with larger body weight or increased weight. Evidence from longitudinal studies also suggests that age‐related bone loss may be lower in obese than nonobese postmenopausal women.^43^ Thus, the role of weight in modifying the PA–bone relationship may be based on several mechanisms, including mechanical loading on the bones and endogenous endocrine factors, particularly sex hormones, adipokines, and inflammatory factors.^18^, ^43^

When we examined associations between the cumulative LTPA score and bone geometry at the midshaft radius from pQCT, we found inconsistent findings in both men and women (Supplemental Table  3). In men, there were negative patterns of associations, albeit nonsignificant, with total and medullary cross‐sectional area at the midshaft radius and total density vBMD at the distal radius, suggesting less endosteal resorption and periosteal expansion in the most active men. Similarly, higher LTPA scores were not associated with bone size in women and associations did not differ by weight, further suggesting that the nonmechanical components of greater fat mass may be driving the PA–density relationship. Together these findings suggest that the potential beneficial effects of LTPA across adulthood in early old age may be achieved by minimizing bone loss, particularly at trabecular‐rich bone sites; however, more data at weight‐bearing sites using 3D‐imaging techniques are required to confirm this.

A key strength of our study is the large population‐based sample of men and women in early old age, with prospective assessment of LTPA at four time‐points over 28 years of follow‐up. This enabled us to relate prospective measures of LTPA to pQCT‐ and DXA‐derived clinically relevant bone outcomes at both non‐weight‐bearing and weight‐bearing sites.

Our study also has several limitations. First, bone outcomes were measured at a single time point in later life; therefore, we are unable to distinguish variation based on the level of peak bone mass achieved by midlife from variation because of subsequent age‐related bone loss. This alongside the observational study design limits our ability to make causal inferences. Second, LTPA was self‐reported and defined as participation in sports, and recreational and leisure‐time activities. This was the focus of our analyses as it is the domain of PA most amenable to intervention, and comparable categories could be identified at each age. However, these measures do not distinguish between load‐bearing and non‐load‐bearing activity, intensity (ie, light, moderate, or vigorous) or level of impact. Nevertheless, the ranking of NSHD participants’ levels of physical activity using self‐reported LTPA measures is similar to data assessed using a combined heart rate and movement‐sensing monitor.^44^, ^45^ Different questions were used to ascertain levels of participation in leisure‐time physical activity at different ages, which is a limitation of our analyses. However, our previous work has shown that the measures at different ages correlate well with each other, other health behaviors, and BMI in expected directions^28^ and are associated with monitored levels of activity at ages 60 to 64.^45^ Third, our findings could be explained by residual confounding. In addition, our analyses were restricted to the sample still alive and participating in the study at ages 60 to 64, and of this sample, we were only able to include those who attended a CRF as this is where pQCT and DXA scans were undertaken. It is possible that additional selection bias may have been introduced because we were unable to include participants who had died or been lost to follow‐up before ages 60 to 64 in our analyses. However, we do not believe this to be a major concern as the study population remains nationally representative in many respects.^46^ Finally, our study population comprised white men and women born in Britain in 1946, which may limit the generalizability of these findings to ethnically diverse or more recently born cohorts. However, the narrow age range of the sample at assessment of bone outcomes limits potential confounding by age, and the findings are likely to be generalizable to the UK population born at a similar time.

In conclusion, this study shows that greater participation in LTPA and maintaining participation across adulthood were associated with higher radial trabecular vBMD and aBMD of the hip and spine in men in early old age. In women, similar patterns of associations were observed with radial trabecular vBMD and total hip aBMD for those who were heavier. Associations with total hip aBMD (in both sexes), and radial trabecular vBMD and spine aBMD (in men) were partly mediated by lean mass. These findings highlight the importance of LTPA across adulthood for the benefit of bone health in old age. That associations were weaker among women suggests that specifıc types of recreational activities that benefit bone health may need to be promoted across adulthood, particularly the premenopausal period, to ensure maximum benefit.

## Disclosures

There are no conflicts of interest to declare.

## Supporting information

Supporting Data.
