# Supplementary material for: Physical Activity Across Adulthood and Bone Health in Later Life: The 1946 British Birth Cohort
Source: J Bone Miner Res. 2018 Dec 10;34(2):252–61. doi: 10.1002/jbmr.3607 (PMC6392181; doi:10.1002/jbmr.3607)
Supplement: Supplementary file 1 — Supporting Data. [file JBMR-34-252-s001.docx]

**Physical activity across adulthood and bone health in later life: the 1946 British birth cohort**

**Supplemental information**

**Supplemental Figure 1:** Percentage differences in a) radius trabecular vBMD and b) hip aBMD by changes in LTPA between ages 36 and 60-64 after adjustment for covariates, by weight among women





**Supplemental Table 1:** Percentage differences in DXA- and pQCT-derived outcomes per 1 unit increase in a cumulative LTPA score adjusted for lean and fat mass indices among men

|  | Radius Trabecular vBMD* | | Radius Total density vBMD | | Total Hip aBMD* | | Spine L1–L4 aBMD | |
| --- | --- | --- | --- | --- | --- | --- | --- | --- |
|  | % diff (95% CI) | p-value for trend | % diff (95% CI) | p-value for trend | % diff ( 95% CI) | p-value for trend | % diff ( 95% CI) | p-value for trend |
| **~~Men~~** |  |  |  |  |  |  |  |  |
| Model 1 | 1.45 (0.66, 2.25) | <.001 | -0.04 (-0.68, 0.61) | .91 | 0.90 (0.42, 1.39) | <.001 | 1.09 (0.53, 1.65) | <.001 |
| Model 2 | 1.50 (0.71, 2.30) | <.001 | 0.004 (-0.64, 0.65) | .99 | 1.10 (0.65, 1.55) | <.001 | 1.20 (0.65, 1.76) | <.001 |
| Model 3 | 1.34 (0.53, 2.14) | .001 | -0.06 (-0.72, 0.59) | .85 | 0.81 (0.37, 1.24) | <.001 | 0.93 (0.39, 1.48) | .001 |

Model 1: height, current smoking status, occupational class and long-term illness

Model 2: model 1 + FMI

Model 3: model 2 + LMI

**Supplemental Table 2:** Percentage differences in DXA- and pQCT-derived outcomes by changes in LTPA between ages 36 and 60-64 (inactive at both ages as the reference category) - adjusted for lean and fat mass indices

|  | **Men** | | | | **Women** | | | |  |
| --- | --- | --- | --- | --- | --- | --- | --- | --- | --- |
|  | **Inactive at 36, active at 60-64** | **Active at 36, inactive at 60-64** | **Active at both ages** | P value* | **Inactive at 36, active at 60-64** | **Active at 36, inactive at 60-64** | **Active at both ages** | P value* | P sex int |
|  | % diff (95% CI) | % diff (95% CI) | % diff (95% CI) |  | % diff (95% CI) | % diff (95% CI) | % diff (95% CI) |  |  |
| **Trabecular radius vBMD^c^**  *N, men=584; women=616* | | | | | | | | | |
| Model 1 ^b^ | -1.26 (-9.82, 7.30) | 3.58 (-1.01, 8.17) | 8.22 (3.44, 13.01) | .003 | -3.06 (-10.5, 4.33) | -0.99 (-6.71, 4.73) | -3.53 (-9.35, 2.29) | .62 | .010 |
| Model 2 ^b c^ | -1.10 (-9.65, 7.45) | 3.30 (-1.30, 7.90) | 8.37 (3.59, 13.15) | .002 | -3.23 (-10.78, 4.30)†  19.5 (-2.68, 41.6)‡ | -2.02 (-7.91, 3.86)†  14.4(-3.06, 31.8)‡ | -2.40 (-8.43, 3.63)†  32.4 (14.5, 50.3)‡ | .82  .005** | .004¶ |
| Model 3 ^b c^ | -2.22 (-10.76, 6.32) | 3.03 (-1.55, 7.60) | 7.46 (2.67, 12.26) | .006 | -3.20 (-10.73, 4.33)†  20.7 (-1.57, 43.0)‡ | -1.79 (-7.68, 4.10)†  15.35 (-2.19, 32.9)‡ | -2.47 (-8.49, 3.56)†  33.3 (15.3, 51.3)‡ | .82  .004** | .002¶ |
|  |  |  |  |  |  |  |  |  |  |
| **Total density vBMD**  *N, men=586; women=617* | | | | | | | | | |
| Model 1 ^b^ | -6.59 (-13.57, 0.39) | -1.54 (-5.27, 2.19) | -2.09 (-5.98, 1.80) | .30 | 2.38(-3.66, 8.43) | -1.13 (-5.78, 3.52) | -1.63 (-6.38, 3.12) | .54 | .33 |
| Model 2 ^b^ | -6.48 (-13.46, 0.49) | -1.73 (-5.47, 2.01) | -2.00 (-5.90, 1.89) | .32 | 4.23 (-1.85, 10.31) | -0.02 (-4.68, 4.64) | 0.39 (-4.46, 5.24) | .48 | .31 |
| Model 3 ^b^ | -6.89 (-13.90, 0.11) | -1.84 (-5.58, 1.91) | -2.33 (-6.26, 1.59) | .26 | 4.29 (-1.79, 10.37) | 0.17 (-4.51, 4.85) | 0.40 (-4.45, 5.64) | .49 | .27 |
|  |  |  |  |  |  |  |  |  |  |
| **Hip aBMD**  *N, men=691; women=769* | | | | | | | | | |
| Model 1 ^b^ | 0.41 (-4.89, 5.72) | 4.59 (1.75, 7.43) | 3.56 (0.62, 6.50) | .009 | -2.54 (-6.34, 1.25) | -0.56 (-3.37, 2.25) | -2.12 (-4.99, 0.76) | .36 | .052 |
| Model 2 ^b^ | 0.64 (-4.37, 5.65) | 3.74 (1.05, 6.43) | 4.10 (1.32, 6.88) | .012 | 0.73 (-2.74, 4.19) | 1.34 (-1.22, 3.90) | 1.87 (-0.81, 4.54) | .55 | .56 |
| Model 3 ^b^ | -1.14 (-5.87, 3.58) | 3.43 (0.90, 5.96) | 2.44 (-0.19, 5.08) | .023 | 0.26 (-3.07, 3.60) | 1.74 (-0.72, 4.20) | 1.17 (-1.41, 3.75) | .52 | .66 |
|  |  |  |  |  |  |  |  |  |  |
| **Spine L1–L4 aBMD**  *N, men=698; women=774* | | | | | | | | | |
| Model 1^b^ | -3.32 (-9.56, 2.91) | 2.55 (-0.78, 5.88) | 3.69 (0.25, 7.13) | .038 | -3.73 (-8.10, 0.64) | -2.06 (-5.33, 1.21) | -1.62 (-4.97, 1.73) | .37 | .14 |
| Model 2^b^ | -3.20 (-9.36, 2.96) | 2.10 (-1.20, 5.40) | 4.00 (0.59, 7.40) | .028 | -1.70 (-5.99, 2.59) | -0.85 (-4.04, 2.35) | 0.89 (-2.44, 4.23) | .54 | .38 |
| Model 3^b^ | -4.85 (-10.83, 1.13) | 1.81 (-1.39, 5.00) | 2.48 (-0.84, 5.81) | .052 | -1.98 (-5.76, 2.80) | -0.60 (-3.76, 2.56) | 0.45 (-2.86, 3.75) | .67 | .42 |

Model 1: height, current smoking status, occupational class and long-term illness; Model 2: model 1 + whole body fat mass index (excl head); Model 3: model 2 + lean mass index

^b^ In women same adjustments as for men plus type and timing of menopause

^c^ in women, interaction between change in LTPA x FMI p ≤.01 so analyses are presented by FMI

**Supplemental Table 3:** Percentage differences in other bone outcomes per 1 unit increase in a cumulative LTPA score

|  | Radius Diaphysis CSA | | Radius Medullary CSA | | Total hip CSA | | Cross-sectional moment of inertia for narrow femoral neck^b^ | |
| --- | --- | --- | --- | --- | --- | --- | --- | --- |
|  | % diff (95% CI) | p-value for trend | % diff (95% CI) | p-value for trend | % diff ( 95% CI) | p-value for trend | % diff ( 95% CI) | p-value for trend |
| **Men** | *N=534* |  | *N=532* |  | *N=638* |  | *N=637* |  |
| Model 1 | -0.17 (-0.68 , 0.35) | .525 | -1.00 (-2.21 , 0.21) | .105 | 0.32 (0.01 , 0.63) | .04 | 0.76 (0.01 , 1.52) | .048 |
| Model 2 | -0.32 (-0.79 , 0.15) | .181 | -1.21 (-2.39 , -0.03) | .044 | 0.20 (-0.05 , 0.46) | .121 | 0.48 (-0.14 , 1.1) | .128 |
| Model 3 | -0.03 (-0.50, 0.45) | .918 | -0.96 (-2.18, 0.26) | .124 | 0.31 (0.05, 0.57) | .019 | 0.65 (0.01, 1.28) | .048 |
| **Women** | *N=600* |  | *N=598* |  | *N=737* |  | *N=737* |  |
| Model 1 | 0.44 (-0.04 , 0.92) ^c^ | .074 | 1.11 (-0.09 , 2.31) | .07 | 0.12 (-0.16 , 0.4) | .39 | -0.48 (-1.20 , 0.24) | .189 |
| Model 2 | 0.40 (-0.05 , 0.85) ^c^ | .083 | 0.75 (-0.47 , 1.97) | .226 | 0.05 (-0.19 , 0.29) | .669 | -0.27 (-0.87 , 0.34)†  0.05 (0.01 , 0.09)‡ | .39  .022** |
| Model 3  ^a^ | 0.45 (-0.02 , 0.92) | .059 | 1.15 (-0.11 , 2.40) | .074 | 0.08 (-0.17 , 0.32) | .53 | -0.22 (-0.85 , 0.40)†  0.05 (0.01 , 0.09)‡ | .49  .015** |
|  |  |  |  |  |  |  |  |  |
| P for sex interaction |  |  |  |  |  |  |  |  |
| Model 1 |  | .09 |  | .015 |  | .35 |  | .06 |
| Model 2 |  | .016 |  | .013 |  | .48 |  | .10¶ |
| Model 3 |  | .022 |  | .017 |  | .38 |  | .09¶ |

Model 1: unadjusted; Model 2: current height and weight; Model 3: model 2 + current smoking status, occupational class and long-term illness

^a^ same model adjustments as for men plus type and timing of menopause

^b^ in women, interaction between LTPA score x weight p ≤.01 so analyses are presented by weight

^c^ LRT test for deviation from linearity p≤.05

† effect estimate for a 1 unit increase in the LTPA score for women of mean weight (72.3kg)

‡ LTPA score x weight interaction term

** p value for LTPA score x weight interaction

¶ p-value for LTPA score x sex x weight interaction
